# The difference in radiographic findings in the distal limbs of working Lipizzan horses, used for dressage or driving

**DOI:** 10.3389/fvets.2024.1393325

**Published:** 2024-05-29

**Authors:** Valentina Zalig, Modest Vengust, Rok Blagus, Dagmar Berner, Cole Sandow, Ashley Hanna, Mitja Miklavcic

**Affiliations:** ^1^Veterina Marc, Sezana, Slovenia; ^2^Veterinary Faculty, University of Ljubljana, Ljubljana, Slovenia; ^3^Faculty of Medicine, University of Ljubljana, Ljubljana, Slovenia; ^4^Royal Veterinary College, London, United Kingdom; ^5^Cole Sandow - Hagyard Equine Medical Institute, Lexington, KY, United States; ^6^Washington State University, Pullman, WA, United States; ^7^UC Davis School of Veterinary Medicine, Davis, CA, United States

**Keywords:** degenerative joint disease, different disciplines, shoeing, studs, working surfaces

## Abstract

**Introduction:**

Lameness originating from the distal limb is common in sport horses and can vary depending on the dynamics of movement and the surface, with differences in shoeing exacerbating this variability. Driving horses work primarily on hard surfaces (pavement), whereas dressage horses work primarily on soft surfaces (riding arenas with sand). Driving horses are traditionally shod with small fixed studs made of hard metal, which are attached to the horseshoe at 4 points, while dressage horses are shod with a simple horseshoe. We investigated the hypothesis that there is a difference in the pathological radiographic findings of the distal limbs between driving and dressage horses. The variability in the stable management and training program was minimized by including horses from the same farm.

**Methods:**

Twenty horses in a driving training program and 20 horses in a dressage program were included in the study. Radiographs of the both front feet were obtained and quantitatively evaluated for radiographic changes by three surgery/diagnostic imaging specialists. Interrater reliability was measured, and multivariate analysis was performed to compare differences in pathological radiographic findings of the distal limbs between the two groups.

**Results:**

Kendal’s concordance coefficient indicated an agreement among raters (Kw ≠ 0) for all observations. Radiographic signs of degenerative joint disease of the distal interphalangeal joint were more common in the group of driving horses compared to dressage horses.

**Conclusion:**

Our hypothesis was confirmed, as there were significant pathological differences between groups in distal articular margin of middle phalanx, joint space narrowing, and irregular joint surface of the middle phalanx.

## 1 Introduction

Lameness originating from the distal aspect of the front limb is common in sport horses in various disciplines and remains one of the main reasons for premature retirement from intensive training and competitions (1–3). Most commonly diagnosed are foot imbalance, bruising, navicular disease, and low-grade degenerative joint disease of the metacarpophalangeal and interphalangeal joints (2, 4).

The nature of the training surface affects the mechanical (over) loading of the distal limbs and plays an important role in the development of lameness (4–6). Horses working on soft, deep surfaces are predisposed to soft tissue injuries caused by mechanical overload. Working on hard surfaces predisposes horses to impact injuries (foot pain, joint trauma, bone bruising) (5–7).

In order to take account of the surface properties and the specific movement dynamics, driving horses are traditionally shod with widia. These are small fixed studs made of hard metal, which are attached to the horseshoe at 4 points. The studs are 5 mm × 12 mm in size, protrude about 5 mm from the shoe and have a conical shape. Dressage horses are shod with a simple shoe without additional grip elements. The use of uniaxial studs interferes with medio-lateral balance and results in abnormal torque forces. The use of biaxial studs could exacerbate the hyperextension of the front fetlocks (8). As the biomechanical properties of the distal limb are dynamically different in both sports (9), we tested the hypothesis that there is a difference in pathological radiographic findings of the distal limbs between driving and dressage horses.

## 2 Methods

### 2.1 Population

All horses in this study were in active training. Twenty horses were used for dressage and 20 horses for driving. All horses were trained 6 times per week for 1–2 h per day. The driving horses were shod with widia and small studs, while the dressage horses were shod with a simple shoe. The trimming and shoeing of the hooves is carried out in consultation with the trainer, farrier and veterinarian and also depends on the level of the training program and the competition season. To ensure consistency, no additional or special measures were included in the farriery program of the individual horses during the research project.

The horses in the dressage group were trained indoors or outdoors on an artificial sandy surface. The horses in the driving group were trained mainly on hard (non-compliant) surfaces. All horses were trained 6 times per week for 1–2 h per day. Driving horses were shod with widia and small studs, while dressage horses were shod with a simple shoe.

The radiographs were acquired during routine examination of horses on the farm. Signed permission was obtained to use the data for this study. The study was approved by the Institutional commission for animal welfare of the Veterinary Faculty, University of Ljubljana (5-5-2020/2).

### 2.2 Radiographic examination

Lateromedial and dorsopalmar projections of both front feet were recorded while the limbs were standing on a wooden block (3 cm thick). The horses stood on both blocks simultaneously, focussing on distal interphalangeal joint (DIP), focal film distance 100 cm. Radiographs were obtained using a digital radiography system (*dicomPACS*®*DX-R*) and a mobile x-ray generator meX + 20BT lite Hybrid power portable X-ray unit and CareView 1500P plate (exposure 72 kV, 1.4 mAs).

### 2.3 Radiographic interpretation

**Figure 1:**
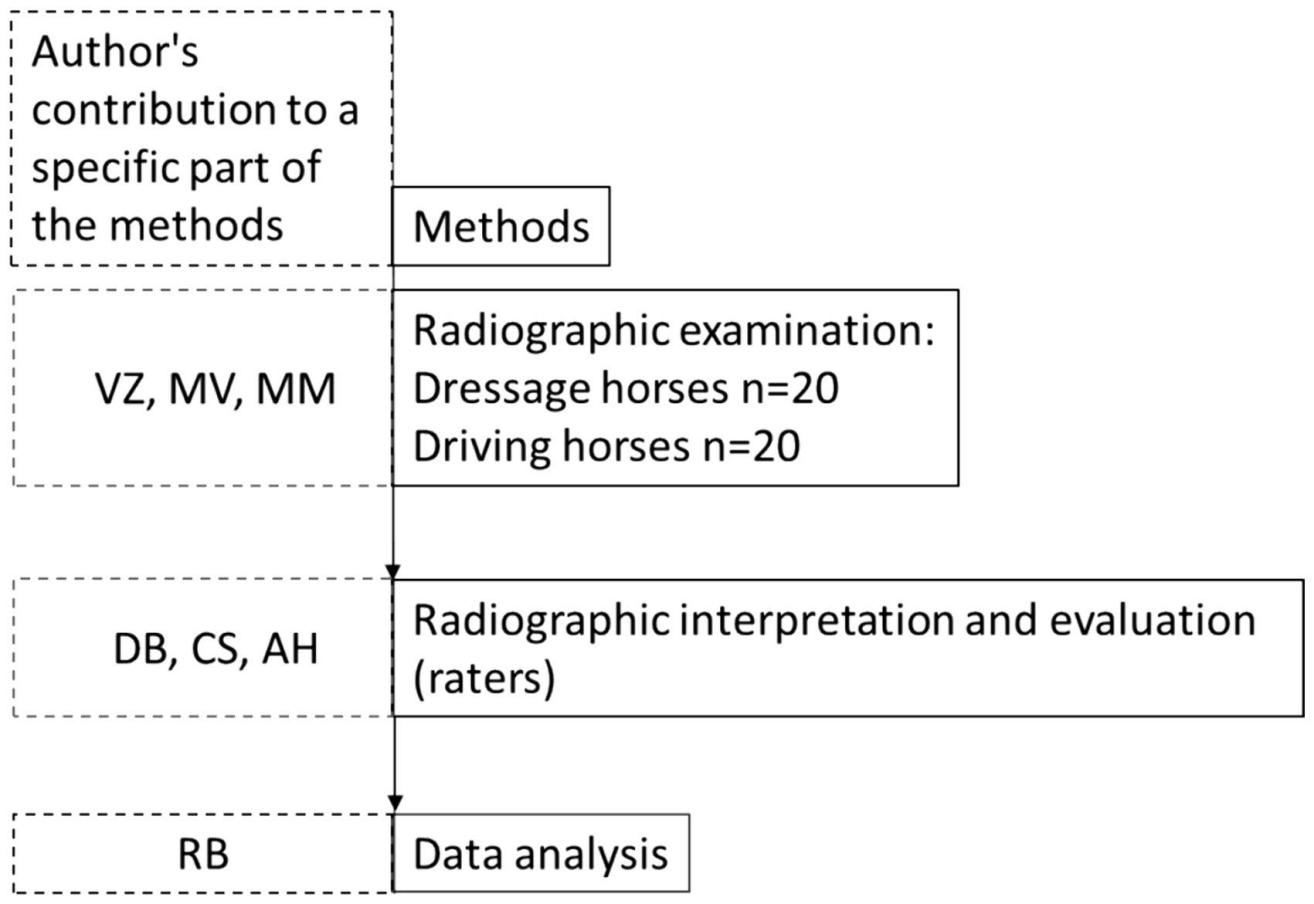
Diagram of the methods. Boxes with solid lines indicate the order of execution of the methods. Boxes with dashed lines indicate the contribution of a particular author to a particular method section. To avoid bias, authors were strictly assigned to a specific method section and did not contribute to the execution of other methods.

Radiographs were evaluated using standard Digital Imaging and Communications in Medicine (DICOM) software, by three board certified specialists, from three different colleges: the American College of Veterinary Radiology (ACVR), European College of Veterinary Diagnostic Imaging (ECVDI), and American College of Veterinary Surgery (ACVS). Radiographic findings were graded according to the grading system defined in Table 1 (10). The observers were blinded as to the origin of the radiographs and the evaluations were performed independently (Figure 1).

**Table 1:** Grading system used for the radiological interpretation [scores were created based on (10)].

1.	Solar angle	1 = <3° 2 = 3–10° 3= >10°
2.	Latero-medial balance	1 = good 2 = poor
3.	Long toe	0 = not present 1 = present
4.	Low heel	0 = not present 1 = present
5.	Distal phalanx rotation	0 = not present 1 = present
6.	Distal phalanx sinking	0 = not present 1 = present
7.	Hoof wall separation (e.g., seedy toe)	0 = not present 1 = present
8.	Outline of the solar margin of the distal phalanx	0 = no irregularity 1 = irregularity 2 = irregularity and modeling
9.	Mineralization in the dermal laminae dorsal	0 = not present 1 = present
10.	Extensor process	0 = normal 1 = mild regular modeling 2 = moderate regular or mild irregular modeling 3 = marked regular or moderate irregular modeling 4 = moderate irregular remodeling
11.	Distal interphalangeal joint (DIP) Proximal articular margin of distal phalanx	0 = normal 1 = mild regular modeling 2 = moderate regular or mild irregular modeling 3 = marked regular or moderate irregular modeling 4 = moderate irregular remodeling 5 = enthesiophytes, present
	DIP joint Distal articular margin of middle phalanx	0 = normal 1 = mild regular modeling 2 = moderate regular or mild irregular modeling 3 = marked regular or moderate irregular modeling 4 = moderate irregular remodeling 5 = enthesiophytes, present
	DIP joint Irregular joint surface	0 = no irregularity 1 = irregularity 2 = irregularity and modeling
	DIP joint Dorsoproximal articular margin of navicular bone	0 = normal 1 = mild regular modeling 2 = moderate regular or mild irregular modeling 3 = marked regular or moderate irregular modeling 4 = moderate irregular remodeling
	DIP joint Joint space narrowing	0 = not present 1 = present
	DIP joint New bone on dorsal diaphysis of the middle phalanx (capsulitis)	0 = not present 1 = present
12.	Osseous changes on DDFT and impar ligament insertion at distal phalanx	0 = normal 1 = mild regular modeling 2 = moderate regular or mild irregular modeling 3 = marked regular or moderate irregular modeling 4 = moderate irregular remodeling 5 = enthesiophytes, present
13.	Palmar process elongation	0 = not present 1 = present
14.	Ungular cartilage ossification	0 = no ossification 1 = mild ossification 2 = moderate ossification 3 = severe ossification 4 = severe asymmetry
15.	Radiolucent zones in middle phalanx	0 = not present 1 = present without articular involvement 2 = present with articular involvement
16.	Radiolucent zones in distal phalanx	0 = not present 1 = present 2 = present with articular involvement
17.	Changes in dorsal margin of distal phalanx	0 = normal 1 = mild regular modeling 2 = moderate regular or mild irregular modeling 3 = marked regular or moderate irregular modeling 4 = moderate irregular remodeling 5 = enthesiophytes, present
18.	Navicular bone	0 = normal 1 = mild regular modeling 2 = moderate regular or mild irregular modeling 3 = marked regular or moderate irregular modeling 4 = moderate irregular remodeling 5 = enthesiophytes, present

### 2.4 Statistical analysis

Age and gender distribution between groups was assessed using Mann–Whitney test with continuity correction and two-sample test for two independent proportions with continuity correction, respectively.

Interrater reliability was measured using Kendal’s coefficient of concordance (Kw), corrected for ties within raters, which tested the null hypothesis of no interrater agreement (Kw = 0). The confidence intervals were obtained using bootstrap: We report adjusted bootstrap percentile intervals based on 10,000 bootstrap resamples obtained with ordinary nonparametric bootstrap.

For the multivariate analysis, the sum of quantitative assessments of all raters was calculated for each outcome. The Poisson model with log link or binary binomial model with logit link were then fitted separately for each of the 22 different outcomes including age, gender and group (dressage was used as a reference category) as independent variables, where only the *p*-value and coefficients for the latter were interpreted. The models were fitted with the generalized estimating equation (GEE) assuming exchangeable working correlation accounting for repeated measurements for each individual (measurements on left and right limbs). The *p*-values for the group effect were adjusted for multiple comparisons with Benjamini-Hochberg method controlling the false discovery rate (FDR) at 10%.

The analysis was performed using R language for statistical computing (R version 4.0.5) (11) Kendall’s coefficients of concordance were calculated using R package DescTools (12) Bootstrap was performed using R package boot (13). GEE models were fitted using R package geepack (14).

## 3 Results

### 3.1 Study population

Forty Lipizzaner horses were included in the study, divided into two equal groups. One group consisted of driving horses (Driving; *n* = 20) and the other of dressage horses (Dressage; *n* = 20). No difference in age or sex distribution was observed between the groups (*p* ≥ 0.45) (Table 2). All of the horses in this study were in active training. The age of the horses ranged from 7 to 25 years.

**Table 2:** Age and gender distribution of all the horses included in the study: mares (F), male horses, geldings, and stallions (M).

AGE (years)	Median	(Q1-Q3)
All horses	11	(8.0–16.0)
Dressage horses	11	(7.7–16.2)
Driving horses	11	(9.7–16)
*p* value	0.5	
**Sex**	**No.**	**%**
Mares (F)	9	22.5%
Males (M)	31	77.5%
Dressage F	3	15.0%
Dressage M	17	85.0%
Driving F	6	30.0%
Driving M	14	70.0%
*p* value	0.45	

### 3.2 Interrater reliability

Kendal’s concordance coefficient indicated an agreement among ratters (Kw ≠ 0) for all observations (Table 3). Mineralization in the dermal laminae was not considered and was not included in the multivariate analysis because it was scored as zero by all three raters in all horses. The confidence interval for distal phalanx sinking and irregular joint surface could not be reported because two of the three raters estimated it to be 0 for all horses.

**Table 3:** Interrater reliability assessment using Kendall’s coefficient of concordance (Kw) with lower (CiL) and upper (CiU) confidence levels.

Test	Kw	CiL – CiU
Solar angle	0.7638	0.6764–0.8371
Lateral medial balance	0.4133	0.3424–0.48
Long toe	0.5248	0.4396–0.6
Low heel	0.265	0.2042–0.3312
Distal phalanx rotation	0.5847	0.3333–1
Distal phalanx sinking	0.3333	N/A
Hoof wall separation	0.62	0.4618–0.7752
Outline of the solar margin of the distal phalanx	0.5176	0.396–0.6492
Extensor process	0.5602	0.4564–0.6654
Proximal articular margin of distal phalanx	0.5524	0.4594–0.6559
Distal articular margin of middle phalanx	0.517	0.4407–0.5931
Irregular joint surface	0.3333	N/A
Dorso-proximal articular margin of navicular bone	0.2987	0.2719–0.317
Joint space narrowing	0.3189	0.2595–0.3333
New bone on dorsal diaphysis of the middle phalanx capsulitis	0.6004	0.5316–0.664
Osseous changes on DDFT and impar ligament insertion	0.344	0.3333–0.39
Palmar process elongation	0.5338	0.4543–0.6043
Ungular cartilage ossification	0.7713	0.6729–0.8454
Radiolucent zones in middle phalanx	0.4601	0.3264–0.6667
Radiolucent zones in distal phalanx	0.4291	0.3137–0.5711
Changes in dorsal margin of distal phalanx	0.5979	0.4765–0.7129
Navicular bone	0.3295	0.2602–0.406

N/A (not applicable): see text for details.

### 3.3 Multivariate analysis for abnormalities in each anatomical section

Results >1 indicate a higher risk ratio (RR) or odds ratio (OR) for the presence of pathologic radiologic findings in driving compared with dressage. The results <1 indicate a lover risk ratio (RR) or odds ratio (OR) for the presence of pathologic changes in driving compared with dressage. The presence of radiographic signs of degenerative joint disease of the distal interphalangeal joint (distal articular margin of the middle phalanx, joint space narrowing, and irregular joint surface) was significantly more common in driving horses. The distal articular margin of the middle phalanx, and irregular joint surface had 95% (*p* value-adj = 0.06) and 153% (*p* value-adj = 0.05) higher outcomes, respectively. Joint space narrowing had four times the odds (*p* value-adj = 0.06) of a higher outcome, meaning it would be more likely observed in the driving horses. Long toe of the front feet have higher outcome in driving horses, with 85% (*p* value-adj = 0.04) (Table 4).

**Table 4:** Multivariate analysis for each anatomical section.

Radiological location	RR/OR	CiL – CiU	*p* value	*p* value-adj
Solar angle	0.97	0.84–1.12	0.68	0.90
Lateral medial balance	0.96	0.84–1.09	0.51	0.83
Long toe	1.85	1.26–2.73	0.00	0.04
Low heel	1.00	0.77–1.28	0.97	0.97
Distal phalanx rotation^*^	4.54	0.57–36.26	0.15	0.56
Distal phalanx sinking^*^	2.19	0.24–19.91	0.49	0.83
Hoof wall separation	1.73	0.72–4.15	0.22	0.63
Outline of the solar margin of the distal phalanx	0.66	0.44–1.00	0.05	0.23
Extensor process	0.92	0.58–1.46	0.73	0.90
Proximal articular margin of distal phalanx	1.28	0.81–2.01	0.29	0.63
Distal articular margin of middle phalanx	1.95	1.16–3.26	0.01	0.06
Irregular joint surface	2.53	1.32–4.83	0.01	0.05
Dorso-proximal articular margin of navicular bone	0.86	0.35–2.07	0.73	0.90
Joint space narrowing^*^	4.08	1.39–11.95	0.01	0.06
New bone on dorsal diaphysis of the middle phalanx capsulitis	0.99	0.73–1.36	0.96	0.97
Osseous changes on DDFT and impair ligament insertion	1.13	0.76–1.66	0.55	0.83
Palmar process elongation	1.12	0.76–1.65	0.57	0.83
Ungular cartilage ossification	0.87	0.67–1.14	0.32	0.63
Radiolucent zones in middle phalanx^*^	4.29	0.36–51.39	0.25	0.63
Radiolucent zones in distal phalanx^*^	0.83	0.14–5.04	0.84	0.93
Changes in dorsal margin of distal phalanx	0.93	0.53–1.63	0.81	0.93
Navicular bone	1.20	0.84–1.70	0.31	0.63

RR, Risk ration for the Poisson model: expected relative difference (in %) of the outcome. OR, odds ratio for the binomial model: the ratio of the odds of having higher/lover outcome between the two groups. ^*^Variables were analyzed using the binary binomial model. CiL – CiU: lower (CiL) and upper (CiU) confidence levels. *p* value-adj: *p* value adjusted for multiple comparisons using Benjamini-Hochberg method.

## 4 Discussion

All horses included in this study were actively working in appointed disciplines and the results reflect subclinical findings. The study was conducted on a population of horses on the same farm with uniform housing, standardized feeding and exercise routines, regular veterinary care and uniform shoeing practises according to their specific sport. Trimming and shoeing are particularly important for the development of orthopedic diseases (15), especially in the distal limb (16, 17).

In this study, several variables were assessed in relation to pathological radiographic findings in the distal limb. Limb loading is greatly influenced by the conformation and position of the limb during stride. Medial-lateral balance, dorso-palmar balance, and break-over are considered the cornerstones of proper foot balance (18), as are the degree of extension of the metacarpophalangeal or metatarsophalangeal joints (19). Our hypothesis was confirmed as this study showed an increased risk for the presence of pathological radiographic findings on the distal limbs in driving horses compared to dressage horses.

The results of a previous study suggest that an increased risk of foot pain is related to breed, type of sport, age and body weight to height ratio, although other factors are also likely to play a role (3). The horses in this study were all of the same breed, and age and sex had no influence on the results of this study. Factors such as the training surface and farriery have an immense influence on the health and longevity of a horse’s career. The phase of rapid loading after initial contact with the surface can be altered by shoeing, training surface and hoof conformation. The hoof is a very effective shock absorber for impact acceleration. Steel shoes increase the amplitude and frequency of the impact, while the use of compliant surface material (such as sand) dampens impact accelerations and forces (20). Working on a hard, non-compliant surface increases the amplitude and frequency of impact accelerations; softer surfaces reduce both. The effects of friction and possible deviating movements must also be taken into account. Soft, compliant surfaces allow the initial heel sink-in during the stance phase, followed by forward rotation of the toe into the surface mid-stance and break over. In contrast, hard surfaces only allow limited rotation of the hoof (20).

Longitudinal sliding of the horse’s hoof may also affect its orthopedic health (21). Horses shod with studs are more stable on hard, non-yielding surfaces, which is critical when pulling a carriage downhill or uphill. The moderate dynamic slip puts less stress on the limb tissue during initial loading (21). Wolff’s law states that bone adapts to the load to which it is subjected; therefore mechanical forces acting on the bone stimulate changes in the bone structure. This is illustrated by increased bone resorption during immobilization or microgravity, and increased bone formation in response to increased mechanical loading (22).

We assessed the outline of the solar margin of the distal phalanx, mineralization in the dorsal dermal laminar interface of the distal phalanx, the distal articular margin of the middle phalanx, dorsoproximal articular margin of the navicular bone, new bone formation at the dorsal diaphysis of the middle phalanx (capsulitis) and impairment of the ligamentous attachment. Articular cartilage damage or loss leading to a narrowing of the joint space of the distal interphalangeal joint usually only becomes apparent in more advanced stages of joint pathology (23). Our results showed that a narrowing of the joint space was more likely in driving horses than in dressage horses, which means that the stress force and strain generated by the specific shoeing and/or the dynamics of movement damage the distal interphalangeal joint more severely in driving horses than in dressage horses.

Pathological radiographic findings in the distal phalanx include osseous cyst-like lesions. They can be found in various locations: (1) adjacent to the articular surface that may communicate with the joint and (2) in the proximal aspect of the distal phalanx. These lesions can usually be recognised on standard radiographs, depending on their size and location (24). In this study, the outlines of the solar margin of the distal phalanx, the distal articular margin of the middle phalanx and the proximal articular margin of the distal phalanx were examined for radiolucent zones. The elongation of the palmar process and changes at the dorsal margin of the distal phalanx (modeling and enthesiophytes) were also assessed. No significant difference was found between the groups in osseous pathology.

The remodeling of the navicular bone and the presence of mineralization in the deep digital flexor tendon as well as the ossification of the hoof cartilage do not appear to be affected differently in dressage and driving horses. In the case of pathological radiographic findings of the navicular bone, this means that peak force and peak load were similar in both groups (25). The navicular bone has a close dynamic and structural relationship with the other structures of the podotrochlear apparatus (the sesamoidean collateral ligaments, the distal sesamoidean impar ligament, the navicular bursa and the deep digital flexor tendon). Chronic stress leads to remodeling of the navicular bone, the altered shape of which can be seen on the lateromedial radiograph. An osteophyte on the dorsoproximal aspect of the navicular bone can probably also affect the distal interphalangeal joint (26). On the other hand, varying degrees of ossification of ungular cartilages are considered normal, also depending on the breed of horse. Extensive ossification of ungular cartilages with a grade of 4 or more may be associated with pain and lameness (27). In most cases, the ungular cartilages are ossified symmetrically. A slightly higher degree of ossification of the lateral cartilage can sometimes be observed, but is usually not clinically significant (10). It is important to note that ossification of the ungular cartilages may have a high heritability, which could also explain the lack of difference between the groups, as the participating horses were all of the same breed (28).

The design of this study reduced the possibility of significant variability between subjects and between raters. Nevertheless, some limitations of the study need to be discussed. Radiological examinations are a routine and frequently used diagnostic procedure for the observation of skeletal changes. For diagnostic purposes, only two radiographs per equine limb were taken in this study, which may limit the diagnostic value. The soft tissue structures within the hoof were also not examined in this study, but this study focused on radiographic changes. The detection of degenerative joint disease by radiographs is also subject to some limitations. Skeletal lesions can only be detected when the mineralization has changed by about 50%. Abnormalities such as early synovitis and cartilage loss are also not recognized on plain radiographs (29).

Another natural limitation of such studies is interrater variability, which is usually more pronounced when raters have a small number of categorization options (1 to 5; Table 1) and even a one-point difference can contribute to higher variability. In general, near-perfect agreement is unlikely in such a study model. However, we rejected the null hypothesis that there is no agreement between raters (i.e.: Kw = 0) and clearly confirmed the alternative hypothesis stating that there is agreement between raters (Kw ≠ 0) (30).

## 5 Conclusion

Radiological signs of degenerative joint disease of the distal interphalangeal joint (pathology in the distal articular margin of the middle phalanx, narrowing of the joint space and irregular joint surface) are more common in the population of active driving horses than in active dressage horses. Driving horses are shod with small studs and widia and work predominantly on hard, non-yielding surfaces, whereas dressage horses are shod with simple shoes and typically work on yielding/compliant surfaces. Therefore, it is possible that the use of friction shoes, which do not allow sliding movement on the ground, are more likely to cause orthopedic problems and potentially shorten the horse’s sporting career. Further research, perhaps including soft tissue pathology, is essential to investigate the individual effects of each insulting variable and determine the clinical significance of our findings.

## Data availability statement

The original contributions presented in the study are included in the article/supplementary material, further inquiries can be directed to the corresponding author.

## Ethics statement

The study was approved by the Institutional commission for animal welfare of the Veterinary Faculty, University of Ljubljana (5-5-2020/2). The studies were conducted in accordance with the local legislation and institutional requirements. Written informed consent was obtained from the owners for the participation of their animals in this study.

## Author contributions

VZ: Conceptualization, Data curation, Formal analysis, Investigation, Methodology, Project administration, Supervision, Visualization, Writing – original draft, Writing – review & editing. MV: Conceptualization, Data curation, Formal analysis, Investigation, Software, Supervision, Validation, Writing – original draft, Writing – review & editing. RB: Data curation, Formal analysis, Software, Validation, Writing – original draft, Writing – review & editing. DB: Conceptualization, Data curation, Formal analysis, Investigation, Supervision, Validation, Writing – original draft, Writing – review & editing. CS: Conceptualization, Data curation, Formal analysis, Writing – original draft, Writing – review & editing. AH: Data curation, Formal analysis, Validation, Writing – original draft, Writing – review & editing. MM: Conceptualization, Formal analysis, Methodology, Project administration, Validation, Writing – original draft, Writing – review & editing.
